# Financial characteristics and security of podiatry work in Victoria: the PAIGE cross sectional study of Australian podiatrists

**DOI:** 10.1186/s13047-023-00657-5

**Published:** 2023-09-16

**Authors:** Anna G. Couch, Terry Haines, Belinda O’Sullivan, Hylton B. Menz, Cylie M. Williams

**Affiliations:** 1 Peninsula Health, Frankston Integrated Health, Allied Health2 Hastings Road, Frankston, VIC 3199 Australia; 2 School of Primary and Allied Health Care, Monash University, 47-49 Moorooduc Hwy, Frankston, VIC 3199 Australia; 3 School of Rural Health, Monash University, VIC, Mercy St, Bendigo, 3550 Australia; 4 The University of Queensland, Brisbane, QLD 4072 Australia; 5 School of Allied Health, La Trobe University, Human Services and Sport, Bundoora, VIC 3086 Australia

**Keywords:** Podiatry, Workforce, Earnings, Finances, Retirement, Rural

## Abstract

**Background:**

Podiatrists’ earnings have an important influence on workforce dynamics. This includes the profession’s ability to attract and retain workers so the population’s healthcare needs can be met. This study aimed to describe financial characteristics of podiatry work and factors relating to a sense of financial security.

**Methods:**

This was a cross sectional study using data from Victorian podiatrists who participated in Wave 1 of the Podiatrists in Australia: Investigating Graduate Employment (PAIGE) survey. Demographic and financial characteristics were described. The outcome measure, financial security, was collected through a self-reported belief based on current financial situation and prospects, respondents’ perception of having enough income to live on when they retire. Univariate logistic regression was used to determine associations with rural or metropolitan practice locations. Multiple ordered logistic regression was performed to explore associations between factors relating to financial security and retirement prospects.

**Results:**

There were 286 Victorian podiatrist (18% of *n* = 1,585 Victorian podiatrists) respondents. Of these, 206 (72% of *n* = 286) identified as female, 169 (59% of 286) worked in the private sector and the mean (SD) age was 33.4 (9.5) years. The mean (SD) annual gross income was $79,194 ($45,651) AUD, and 243 (87% of 279) made regular superannuation contributions. Multiple ordered logistic regression analyses identified factors associated with podiatrists’ perception of having adequate retirement income. These included being an owner/partner of their main workplace (adj OR = 2.70, 95% CI = 1.49–4.76), growing up in a rural location (adj OR = 2.27, 95% CI = 1.38–3.70), perceiving a moderate overall health rating (adj OR = 2.03 95% CI = 1.51–2.75), not having financial debt related to education and training (adj OR = 2.02, 95% CI = 1.24–3.32) and regular contributions to a superannuation scheme (adj OR = 4.76, 95% CI = 2.27–10.00).

**Conclusion:**

This is the first known study to explore podiatrists’ earnings and perceptions regarding financial security. Findings suggest modifiable ways to improve financial security of podiatrists including support and education about personal and business finances including debt management, understanding the importance of contributions to superannuation when self-employed, and developing skills and supports for podiatrists to run their own businesses. This research is exploratory and is relevant for understanding the impact that income and financial security have on workforce dynamics.

**Supplementary Information:**

The online version contains supplementary material available at 10.1186/s13047-023-00657-5.

## Background

Allied health comprises the largest clinical workforce in primary health care in Australia, however access to allied health workers is inequitable with shortages in rural and remote areas [1]. Podiatrists’ earnings have an important influence on workforce supply and sustainability, including the attraction and ability of the workforce. These dynamics ensure the healthcare needs of all Australians can be met. Yet little is known about earnings and debt within the profession in Australia or more broadly in the international podiatry community.

Podiatry has been described as a career that offers diversity in workload, opportunities to upskill, flexible working arrangements and excellent employability in public and private settings [2, 3]. Earning capacity is related to these settings, and remuneration methods (either salaried or contracted) and bargaining agreements can impact workforce dynamics. In Australia, the taxation law outlines the differences between employees and contractors [4]. Therefore, salaried employees are remunerated based on their award or negotiated employment arrangement, whereas contracted podiatrists are self-employed and take an agreed amount and/or a percentage of income from patient fees. This secondary model requires sufficient throughput or services of different client types to generate adequate fee-for-service income. Salaried employment arrangements include access to leave (paid and unpaid) as well as minimum mandatory contributions paid by the employer to a superannuation scheme as set out by the Australian Government [5]. In contrast, podiatrists who are contracted typically work as sole traders and are responsible for making their own contributions to a superannuation scheme [6], and do not always have access to leave entitlements. A recent study of Australian podiatrists identified that 16% (180 of 1,129) work in these contracted arrangements and 51% (573 of 1,129) are salaried employees [7].

Little is known about podiatrist remuneration and any relationship to workforce dynamics, but it is hypothesised that income could impact workforce dynamics. In a recent qualitative study of six student podiatrists exploring the question “why study podiatry?” [2], one participant focused on remuneration. Another recent study within the United Kingdom identified financial pressures related to the removal of the National Health Service bursary, financial support provided by the United Kingdom government, being a key deterrent for students choosing podiatry as a career [8]. Whilst these studies explored motivations and barriers towards podiatry as a career, the impact of debt related to education is a workforce dynamic consideration.

In Australia the average retirement age is 55 years [9], but the age at which people are eligible to access the government funded age pension is 66 years and 6 months [10]. This access to pension funds is set to increase to 67 years from 1^st^ July 2023 [10]. The average age that podiatrists retire in Australia is unknown, but currently 18% of the Australian podiatry workforce are aged between 50–64 years and 2% are aged over 65 years [11]. In a study of nurses, financial security was found to be associated with early retirement [12] and intent to leave was linked to ‘maximum superannuation benefits reached’ and ‘optimal taxation situation’ [13]. Whilst data exists in the nursing profession, most of whom work in salaried arrangements, it may not be transferable to allied health professions with different training pathways, training subsidies, employment structures and awards through which they are remunerated.

The 10 year Primary Care Plan [1] and the Strengthening Medicare Taskforce Report [14] identifies the need to fast track work to improve the supply and distribution of allied health professionals including podiatrists as a key component of health prevention and primary care system development. In the current economic climate, it is timely to consider financial security and its predictors as a potential component impacting podiatry workforce dynamics. Therefore, the primary aim of this study was to describe the financial characteristics of podiatry work and the factors relating to a sense of financial security.

## Methods

### Study design

This study used cross-sectional data from the first wave of the Podiatrists in Australia: Investigating Graduate Employment (PAIGE) survey collected in 2017. The PAIGE study was designed to explore different elements of the podiatry workforce to understand recruitment and retention. Approval for PAIGE was provided by the Monash University Human Research Ethics Committee (7871). Details of the PAIGE study methods are published in detail elsewhere [15]. The researchers used the CHERRIES (Checklist for Reporting Results of Internet E-Surveys) to guide collected data reporting [16].

### Participants and setting

All podiatrists and podiatric surgeons working in Victoria, Australia, were invited to participate in Wave 1 of the survey. The survey was open between 21^st^ of February 2017 to 27^th^ September 2017. When the survey closed, there were an estimated 1,585 podiatrists registered in Victoria [17]. Participants were recruited through direct emails to podiatry alumni university graduate lists, the Australian Podiatry Association, at Victorian podiatry conferences and via social media accounts of the research team (Facebook, LinkedIn, and Twitter). Podiatrists were also encouraged to share the survey link with podiatry colleagues. Participants were given the option at the end of the survey to leave their contact details to participate in the draw for one of five $100AUD Coles Myer gift cards, participate in a focus group, and receive results following the survey closure.

### Data collection

The PAIGE survey questions were based on the Medicine in Australia: Balancing Employment and Life (MABEL) study [18]. Data were collected as per the PAIGE study methodology [19] and details related to data domains collected in each wave are published elsewhere [15]. Wave 1 survey is provided as Supplementary File 1.

Demographic data collected in Wave 1 included information about age, gender, years practicing, employment profile, rural background, and overall health rating. Data relating to podiatrists’ finances included information about gross earnings, gross household income, whether they received any ‘in kind’ benefits or subsidies as part of their employment, debt related to podiatry education and training (including HECS debt or other debt associated with podiatry training expenses), financial investment in private practice, superannuation contributions, and professional medical liability and insurance premiums. Participants were asked to indicate their level of agreement as to whether they would have enough to live on when they retire through a 5-point Likert scale (1 = *strongly agree*, 2 = *agree*, 3 = *neutral*, 4 = *disagree*, 5 = *strongly disagree*) [20].

### Procedure

Survey data were collected through Qualtrics® software (Qualtrics, Provo, UT, USA) [21]. Participating podiatrists were asked to create their own unique identifier code so that subsequent wave responses could be linked. Podiatrists could withdraw at any time by closing their internet browser and forced or requested prompts were used to minimise missing data. Due to the sensitive nature of financial information, questions in this section of the survey were optional and podiatrists could answer all, some or none of the financial questions and still progress through the survey. Cookies were used to save responses for up to four hours for partial completion and Internet Protocol (IP) addresses are routinely collected by Qualtrics® as the de-identified metadata in the survey responses.

### Analysis

**Fig. 1:**
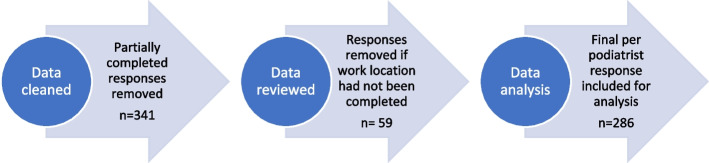
Summary of data analysis

Data were initially cleaned to remove any responses not including core demographics (age, gender, recency of practice, work setting, postcode where the participant lived). These data points were deemed essential for the broader research aim of the PAIGE study. Data were reviewed and further cleaned if podiatrists had not provided the suburb and postcode of their work location. Data management and cleaning decisions are outlined in Fig. 1. Data were analysed using Stata 15 software (StataCorp, College Station, TX, USA). Descriptive statistics of all variables of interests were grouped for the entire cohort and then categorised based on postcode data into metropolitan responses (MMM 1) or rural responses (MMM 2, 3, 4, 5, 6 and 7) using the Modified Monash Model (MMM) [22]. This grouping method was used to explore contextual differences. Likert scale data relating to agreement was reduced to a 3-point scale (Agree (combined Strongly Agree and Agree), Neutral, Disagree (combined Strongly Disagree and Disagree)). Univariate regression analysis was used initially to determine if demographic and financial/remuneration factors were associated with work location (metropolitan or rural). Subsequently, data from all podiatrists regardless of working location was combined and multivariate analysis was applied to explore factors associated with financial situation and prospects, the perception they will have enough to live on at retirement. The multivariate model was based on backwards stepwise multiple ordered logistic regression. Factors within this model were chosen where univariate analysis revealed a value of *p* ≤ 0.20. The variable with the least significant fit was then removed in a backward stepwise procedure until all remaining variables were significant at *p* < 0.05. The research team built both the univariate and multivariate model based on variables known to impact financial security from the MABEL study [18]. Results are reported in Odds Ratios (OR), adjusted Odds Ratios (adj OR) and 95% confidence intervals (95% CI).

## Results

### Participant characteristics

Two hundred eighty-six podiatrists who completed most of the demographic questions in Wave 1 of the survey. When the Wave 1 survey closed, the response rate was estimated to be 18% (286 of 1,585) of registered Victorian podiatrists [17]. Table 1 outlines a summary of podiatrists’ demographics, work setting, employment profile, rural background, and health rating. Of the total responses (*n* = 286), 206 (72%) identified as female and mean (SD) age of podiatrists were 33.4 (9.5) years. For over half (59% of *n* = 286) the primary work setting was within the private sector, 170 (59%) podiatrists were salaried employees, and the mean (SD) number of working locations was 2.2 (1.7). The mean (SD) time working in this location was 5.6 (6.9) years and 108 (38%) of podiatrists grew up in a rural location. Of the total number (*n* = 286) of podiatrists, 119 (42%) deemed their health as ‘excellent’, 113 (39%) ‘very good’ and 47 (16%) as ‘good’.

**Table 1:** Demographics of participants

	**Mean (SD)** **Frequency (%)** ***n* = 286**	**Metro** **Mean (SD)** **Median (IQR**^a^**)** ***n* = 234**	**Rural** **Mean (SD)** **Median (IQR**^a^**)** ***n* = 52**	**OR, 95% CI**
**Age**	*n* = 285	*n* = 234	*n* = 51	
(years)	33.4 (9.5)	33.3 (9.1)	34.0 (10.9)	0.99 (0.96–1.02)
**Gender**	*n* = 286	*n* = 234	*n* = 52	
Did not identify as female	80 (28%)	65 (28%)	15 (29%)	
Female	206 (72%)	169 (72%)	37 (71%)	1.05 (0.54–2.05)
**Recency**	*n* = 286	*n* = 234	*n* = 52	
(years)	9.9 (9.2)	9.8 (9.1)	10.1 (9.8)	0.99 (0.97–1.03)
**Primary work setting**	*n* = 286	*n* = 234	*n* = 52	
Private practice	169 (59%)	132 (56%)	37 (71%)	
Public health service	117 (41%)	102 (44%)	15 (29%)	1.91 (0.99–3.66)
**Business relationship**	*n* = 284	*n* = 231	*n* = 52	
Owner or partner	65 (23%)	49 (21%)	16 (31%)	Reference value
Salaried employee	170 (59%)	141 (60%)	29 (56%)	1.59 (0.80–3.17)
Contracted employee	48 (17%)	41 (18%)	7 (13%)	1.91 (0.72–5.10)
**Working locations**	*n* = 285	*n* = 234	*n* = 51	
(number)	2.2 (1.7)	2.2 (1.7)	2.3 (1.5)	0.96 (0.81–1.14)
**Time working in this location**	*n* = 286	*n* = 234	*n* = 52	
(years)	5.6 (6.9)	5.3 (6.8)	6.6 (7.4)	0.70 (0.32–1.52)
**Grew up in a rural area**	*n* = 286	*n* = 234	*n* = 52	
(yes)	108 (38%)	76 (32%)	32 (62%)	0.30 (0.16–0.56)
**Overall health rating**	*n* = 286	*n* = 234	*n* = 52	
Excellent	119 (42%)	106 (45%)	13 (25%)	Reference value
Very good	113 (39%)	86 (37%)	27 (52%)	0.39 (0.19–0.80)
Good	47 (16%)	37 (16%)	10 (19%)	0.45 (0.18–1.12)
Fair	7 (2%)	5 (2%)	2 (4%)	0.31 (0.05–1.74)
Poor	0 (0%)	0 (0%)	0 (0%)	-

^a^Interquartile range

### Finances

There were 212 (74% of 286) podiatrists who provided detailed responses about their finances (Table 2). The average annual gross earnings (before tax) were $79,194 ($45,651) AUD and the annual gross household income was $153,240 ($94,300) AUD. Of the total number of podiatrists, 14 (5% of *n* = 286) received ‘in kind’ benefits or subsidies as part of their job and the median (interquartile range) was $5,000 ($2,000 to $10,000) AUD. There were 112 podiatrists (39% of 286) who indicated having a debt and the mean (SD) debt was $36,629 ($44,869). A quarter of podiatrists (71 of 286) indicated a financial investment in a private podiatry practice with the mean (interquartile range) amount owing of $109,555 ($12 to $779) AUD. There were 243 (87% of 279) podiatrists indicating they (or their employer) regularly contributed to a superannuation scheme, and 96 (33% of 286) agreeing with the statement “Given my financial situation and prospects, I believe I will have enough to live on when I retire”. The mean (SD) amount that podiatrists paid for professional medical liability or insurance premiums was $600 ($1,911) AUD.

**Table 2:** Participant financial characteristics

Financial characteristics	Total responses N (%) Mean (SD) Median (IQR^a^) *n* = 286	Metro N (%) Mean (SD) Median (IQR^a^) *n* = 234	Rural N (%) Mean (SD) Median (IQR^a^) *n* = 52	OR, 95% CI
**Gross earnings (before tax)**	*n* = 212	*n* = 173	*n* = 38	
Annual ($)	79,194 (45,651)	80,340 (47,531)	72,372 (35,047)	1.00 (0.99–1.00)
**Gross household income (before tax)**	*n* = 172	*n* = 122	*n* = 24	
Annual ($)	153,240 (94, 300)	156,980 (100,051)	130,196 (52,442)	1.00 (0.99–1.00)
**‘In kind’ benefits**	*n* = 286	*n* = 233	*n* = 52	
Yes	14 (5%)	12 (5%)	2 (4%)	0.74 (0.16–3.40)
($)	5000 (2000–10,000)	5000 (2250–10,000)	7725 (450–15,000)	
**Education/training financial debt**	*n* = 286	*n* = 233	*n* = 52	
Yes	112 (39%)	94 (40%)	18 (35%)	Reference value
Unsure/prefer not to say	52 (18%)	39 (17%)	13 (25%)	0.57 (0.26–1.29)
No debt	122 (43%)	100 (43%)	21 (40%)	0.91 (0.46–1.82)
**Education/training financial debt**	*n* = 112	*n* = 94	*n* = 18	
($)	36,629 (44, 869)	31,957 (23,017)	54,805 (98, 909)	0.99 (0.99–1.00)
**Financial investment in private practice**	*n* = 286	*n* = 233	*n* = 52	
Yes	71(25%)	55(24%)	15 (29%)	1.31 (0.67–2.57)
**Total financial debt from owning private practice**	*n* = 28	*n* = 20	*n* = 7	
($)	109,555 (125, 779)	93,277 (110, 633)	170,286 (159, 981)	0.99 (0.99–1.00)
**Status of private practice for tax**	*n* = 71	*n* = 55	*n* = 15	
Sole trader	29 (41%)	24 (44%)	5 (33%)	Reference value
Partnership	5 (7%)	3 (5%)	2 (13%)	0.31 (0.04–2.38)
Company	21 (30%)	16 (29%)	4 (27%)	0.83 (0.19–3.58)
Trust	16 (22%)	12 (22%)	4 (27$%)	0.63 (1.14–2.76)
**You (or employer) regularly contribute to superannuation scheme**	*n* = 279	*n* = 227	*n* = 51	
Yes	243 (87%)	198 (87%)	44 (86%)	1.09 (0.45–2.64)
**“Given my current financial situation and prospects, I believe I will have enough to live on when I retire”**	*n* = 286	*n* = 233	*n* = 52	
Agree	96 (33%)	76 (32%)	19 (36%)	Reference value
Neutral	82 (29%)	67 (29%)	15 (29%)	1.12 (0.53–2.37)
Disagree	108 (38%)	90 (39%)	18 (35%)	1.25 (0.61–2.55)
**Professional medical liability/insurance premiums in last 12 months**	*n* = 188	*n* = 149	*n* = 38	
** (**$)	602 (1,911)	472 (738)	1100 (3,994)	0.99 (0.99–1.00)

^a^Interquartile range

### Job characteristics and finances related to geographical variants

Univariate analysis showed there were significant differences related to demographics and work location, but no significant differences related to finances and work location (Tables 1 and 2). Podiatrists who worked in metropolitan locations were less likely to have grown up in a rural area (OR = 0.30, 95% CI = 0.16–0.56). Podiatrists who worked in metropolitan locations were less likely to regard their health as ‘very good’ (OR = 0.39, 95% CI = 0.19–0.80) compared to podiatrists working in rural locations. There were no significant differences between gross annual earnings, debt for education or training or debt related to owning a private practice between podiatrists working in each location.

### Current financial situation and impact on retirement

Once all podiatrists were combined, rather than separating participants by rural and metropolitan location, multivariate analysis demonstrated several significant factors relating to podiatrists who agreed with the statement “Given my current financial situation and prospects, I believe I will have enough to live on when I retire” (Table 3). Multiple ordered logistic regression analyses indicated that podiatrists with a higher level of agreement owned or were a partner of their main workplace (adj OR = 2.70, 95% CI = 1.49–4.76); grew up in a rural location (adj OR = 2.27, 95% CI = 1.38–3.70); perceived a moderate rating of overall health (adj OR = 2.03 95% CI = 1.51–2.75); did not have financial debt related to their education and training (adj OR = 2.02, 95% CI = 1.24–3.32); and they or their employer regularly contributed to a superannuation scheme (adj OR = 4.76, 95% CI = 2.27–10).

**Table 3:** Factors impacting podiatrists’ perception on whether they will have enough money to live on when they retire

“Given my current financial situation and prospects, I believe I will have enough to live on when I retire”	Odds Ratio (OR)	95% CI	*p*
Owned or were a partner of their main workplace	0.37	0.21–0.67	0.001
Grew up in a rural location	0.44	0.27–0.72	0.001
Perceived moderate rating of overall health	2.03	1.51–2.75	< 0.001
Did not have a financial debt related to their education and training	2.02	1.24–3.32	0.005
They or their employer regularly contributed to a superannuation scheme	0.21	0.10–0.44	< 0.001

## Discussion

This is the first known study to explore podiatrists’ financial characteristics and perceptions regarding financial security as a potential factor related to workforce dynamics, including overall workforce attraction and retention. This research is fundamental to building solutions to ensure workforce sustainability in the podiatry profession. If podiatrists perceive they don’t have adequate financial income and secure prospects for more income, relative to debt accrued from studying and the costs of running a business, they may choose to retrain or seek employment outside of direct clinical care. How podiatrists are remunerated and employed is modifiable, often through awards and mutual negotiation through the employer and employee/sub-contractor. Therefore workforce planners should strongly consider the findings within this study to address business support, superannuation, and debt reconciliation These factors have potential to impact workforce dynamics.

The association between regular contributions to a superannuation scheme and participants agreeing that they will have enough to live on when they retire is unsurprising. Superannuation contributions in Australia has been compulsory for employers since 1992 and has been legislated to rise incrementally each year until it reaches 12% in 2025 [23]. However, a subset of podiatry positions are contractual in nature such that podiatrists are sole traders for tax purposes and therefore individually responsible for making their own superannuation contributions [6]. The Australian Tax Office has identified the challenges with this business model for business sustainability (25% Australian workforce is self-employed contractors working under this arrangement) [24]. Regarding the podiatry profession, the policy regarding superannuation for contractors is a key factor for self-employed podiatrists preparing for retirement. Lower superannuation contributions over a working life would mean lower superannuation balances at retirement and the need to continue working for more years [24]. Overall, this may underpin the finding that 38% of the podiatrists in this study did not perceive financial readiness for future retirement.

This study provides insight into educational debt and earnings related to podiatry. Podiatrists who did not have debt related to education and training were more likely to agree they would have enough to live on when the retired. In the Australian context, government subsidised loans are available to pay for study when podiatrists are enrolled with an approved higher education provider [25]. The government financial support for allied health degrees in Australia is provided to education providers at a fixed rate, which has received little increases in the last 20 years. This has resulted in universities having increasing course fees to remain viable [26]. 2021 saw the most recent impact on university fees in for health care education. This impact saw a change in Commonwealth Supported Places, where students paid a reduced fee or acquired a smaller debt compared to previous years [26]. These changes saw the greatest impact on nursing, with limited changes to medical and allied health education [26]. Further steps to support nursing training were taken by some Australian state and territory governments as a result of the COVID-19 pandemic. Most recently, the Victorian Government implemented a bursary of $9000 for enrolled nursing students, and an additional $7500 on graduation. This financial contribution would clear any enrolment debt if a nurse went on to work in the public health system for two years after finishing their course from 2023 [27]. Few bursaries are available to podiatry students in Australia and completion of a pre-registration degree will cost a student between $33,000-$120,000. Subsidies for allied health education have been shown to be successful in increasing university intake numbers in the United Kingdom [8].

This study further highlights that skills in business management and viability are a key factor related to financial security of podiatrists. Podiatrists are trained in tertiary institutions to deliver podiatry care and there is limited compulsory curriculum in how to run a profitable business. Podiatrists who run a business may have to obtain skills and qualifications through professional associations, tertiary institutions, or government education programs. At the time of data collection, the average wage podiatrists earned was consistent with the average Victorian wage, and greater than the average health worker wage [28], highlighting the earning potential for podiatrists. Despite podiatrists reported earnings, education investment and practice debt podiatrists appear to accrue, workforce planners should consider partnering with education providers to build business skills for podiatry students including how they enable superannuation, HECS-HELP debt repayments, ensure leave provisions and manage profit, loss and long-term business growth, as factors related to sustainable financial security of podiatrists. Peak bodies may also consider providing support to new graduates in business and financial literacy. This is a key recommendation set out by the Rural Health Commissioner as a potential enabler of allied health capacity building to support service access in rural locations [29].

When interpreting the findings of this study, the limitations should be considered. Whilst the data provides an accurate representation of the Victorian podiatry workforce, results may not be generalisable to the entire Australian podiatry workforce. The data was also collected prior to the COVID-19 pandemic, in a time of relative economic security. This means it is important to consider what the findings would look like now if they were collected again following the pandemic with low growth and lower numbers of students than in the past [30]. As this was a singular cross-sectional study design, the research team investigated the predictor variable and outcome measure of podiatrists at the same time and therefore it is difficult to derive causal relationships between actual retirement and podiatrists leaving the profession [31].

Attitudes towards financial security can change over time and are based on significant external factors therefore, further research is required to explore podiatrists’ perceptions regarding their financial status and its impact on retention within the profession. A mixed-method approach using longitudinal data would provide more comprehensive information in both stable and unstable economies about how podiatrists perceive their financial security and how it impacts workforce decisions.

## Conclusion

This study is the first to explore financial characteristics and financial security of podiatry work. In 2017, Victorian podiatrists’ average annual gross income was $79, 194, with 36% of podiatrists having $36, 629 average debt related to education or training. Financial security, as indicated by belief that podiatrists would have enough to live on when they retired, was related to being an owner or partner of a practice, having grown up in a rural location, not having debt related to education or training and having access to regular contributions into superannuation. Workforce planners and the Australian Government should strongly consider strategies around debt reconciliation, business management support and superannuation to enable financial security in the profession as a possible factor influencing workforce dynamics Further research could assist in understanding how earnings and debt impact intent to leave the profession in both stable and unstable economies.

### Supplementary Information

**Additional file 1.** 

## Data Availability

Request for further details of the PAIGE data set and queries relating to data sharing arrangements may be submitted to Cylie Williams (cylie.williams@monash.edu). Aggregate or summarised data may be shared based on reasonable request.

## Abbreviations

PAIGE: Podiatrists in Australia: Investigating Graduate Employment
NHS: National Health Service
MABEL: Medicine in Australia Balancing: Employment and Life
IP: Internet Protocol
COVID-19: Coronavirus pandemic
